# Standardising DVLA Driving Guidance at Discharge for Psychiatric Inpatients

**DOI:** 10.1192/bjo.2026.11381

**Published:** 2026-06-30

**Authors:** Evangelos Galatis, Mehr Usman

**Affiliations:** North Staffordshire Combined Healthcare NHS Trust, Stoke-on-Trent, United Kingdom

## Abstract

**Aims:**

The DVLA (Driver and Vehicle Licensing Agency) rules require patients with psychiatric diagnoses such as psychosis, mania, or depression to stop driving and/or notify the DVLA. Despite this, patients are often discharged without being informed of these legal responsibilities. There was no standardised approach within our trust to ensure this guidance was communicated clearly and consistently.

This project aimed to improve the communication of DVLA driving restrictions to psychiatric inpatients being discharged on medications or with a diagnosis that may impair fitness to drive.

**Methods:**

We created clear, patient-friendly DVLA information leaflets, covering psychosis, mania/hypomania, and depression, based directly on the latest DVLA guidance. These were uploaded to the Trust resource page. A prompt was also added to the electronic discharge summary to remind clinicians to check that the relevant guidance has been provided.

**Results:**

The leaflets provided a simple, visible solution that improved staff confidence in advising patients. Feedback from members of the team highlighted that easy access to up-to-date, printable resources removed uncertainty around DVLA requirements. Discharge summaries more frequently included DVLA advice, and patients were consistently provided with relevant written information.

**Conclusion:**

Introducing guidance leaflets within the trust enabled a sustainable, straightforward solution to a long-standing gap in discharge communication. This project improved DVLA compliance, patient safety, clinician confidence, and could be easily replicated across other wards and trusts. We plan to expand this project to other mental health services, such as the Crisis Resolution and Home Treatment Team (CRHTT). This will include adapting the existing leaflets to suit community and crisis settings, ensuring patients consistently receive appropriate DVLA advice across all points of care.

